# A Comparison of Microscale Techniques for Determining Fracture Toughness of LiMn_2_O_4_ Particles

**DOI:** 10.3390/ma10040403

**Published:** 2017-04-12

**Authors:** Muhammad Zeeshan Mughal, Hugues-Yanis Amanieu, Riccardo Moscatelli, Marco Sebastiani

**Affiliations:** 1 Engineering Department, University of Rome “ROMA TRE”, Via della Vasca Navale 79, 00146 Rome, Italy; riccardo.moscatelli@uniroma3.it (R.M.); marco.sebastiani@uniroma3.it (M.S.); 2 Institute for Materials Science and Center for Nanointegration Duisburg-Essen (CENIDE), University of Duisburg-Essen, Universitätsstr. 15, 45141 Essen, Germany; hy.amanieu@uni-due.de or HuguesYanis.Amanieu@leclanche.com; 3 Robert Bosch GmbH, Robert-Bosch-Platz 1, 70839 Gerlingen-Schillerhoehe, Germany

**Keywords:** fracture toughness, atomic force microscopy, pillar splitting, lithium-ion batteries, nanoindentation, focused ion beam

## Abstract

Accurate estimation of fracture behavior of commercial LiMn_2_O_4_ particles is of great importance to predict the performance and lifetime of a battery. The present study compares two different microscale techniques to quantify the fracture toughness of LiMn_2_O_4_ particles embedded in an epoxy matrix. The first technique uses focused ion beam (FIB) milled micro pillars that are subsequently tested using the nanoindentation technique. The pillar geometry, critical load at pillar failure, and cohesive FEM simulations are then used to compute the fracture toughness. The second technique relies on the use of atomic force microscopy (AFM) to measure the crack opening displacement (COD) and subsequent application of Irwin’s near field theory to measure the mode-I crack tip toughness of the material. Results show pillar splitting method provides a fracture toughness value of ~0.24 MPa.m^1/2^, while COD measurements give a crack tip toughness of ~0.81 MPa.m^1/2^. The comparison of fracture toughness values with the estimated value on the reference LiMn_2_O_4_ wafer reveals that micro pillar technique provides measurements that are more reliable than the COD method. The difference is associated with ease of experimental setup, calculation simplicity, and little or no influence of external factors as associated with the COD measurements.

## 1. Introduction

Low cost and low toxicity of spinel LiMn_2_O_4_ makes them a good cathode material for the lithium-ion batteries. Unfortunately, its commercialization is limited by its short lifetime. The primary reason for its short lifetime is extensively documented and related to the dissolution of manganese atoms in the electrolyte, which is the main source for capacity fade [1,2]. Mechanical failure upon cycling produces more surface area that could lead to the loss of material through dissolution. It can also result in the loss of adhesion with the current collector [3]. Internal pressure associated with the intercalation and deintercalation results in the fracture of the crystal. It was previously reported for LiMn_1.95_Al_0.05_O_4_, also a spinel that two parallel phenomena occurs: (1) brittle cracking at the first electrochemical cycle and (2) fatigue leading to fracture [4]. More recently, the same issues were observed for commercial LiMn_2_O_4_, where oxygen deficiency is witnessed; a phenomenon which can be reduced with stoichiometric spinel [5]. It has been shown that the favorite cracking plane is {111} [6]. It is expected for LiMn_2_O_4_ as {111} planes have the lowest solid-to-vapor surface energy, but {101} faceting occurs as well [6]. The main challenge associated with the fracture toughness measurement of LiMn_2_O_4_ spinal materials is that a bulk single crystal of this type of spinel cannot be easily grown above a few micrometers [7]. Therefore, it is difficult to measure the fracture properties of single grains without the use of microscale techniques. 

Fracture toughness measurement using the indentation testing has been widely used over the past three decades for brittle materials such as glasses and ceramics [8,9,10,11]. Lawn et al. [8] (and then Anstis et al. [9]) suggested the classic relationship of fracture toughness assessment using Vickers indentation based on the half-penny crack configuration

(1) $$K_{c}=\alpha \;{\left(\frac{E}{H}\right)}^{1/2}\frac{P}{c^{3/2}}$$

where *P* is the indentation load, *c* is the radial crack length from indentation center to the crack tip, *E* is the Young’s modulus, *H* is the hardness, and *α* is the constant that depends upon indenter geometry. Anstis et al. empirically determined the value of α as 0.016 ± 0.004 for Vickers indentation [9]. With the development of nanoindentation testing in the early 1990s [12], it was revealed that Equation (1) also applies to the three sided Berkovich indenter commonly used in the nanoindentation testing. Later Jang and Pharr [13] suggested that the indenter angle has an effect on the cracking behavior and can influence the fracture toughness values. Their study using Si and Ge shows that by simply changing the indenter shape from cube corner (35.3°) to Berkovich (65.3°) indenter, the coefficient value decreases by ~50%. 

In the present article, we compare two microscale techniques, namely pillar splitting method [14] and crack opening displacement (COD) [15], to characterize the fracture toughness values of micrometric particles of LiMn_2_O_4_. In the first technique, nanoindentation is used to split the focused ion beam (FIB) milled micro pillars. Fracture is realized by splitting at reproducible loads that are experimentally quantified from displacement bursts in the loading segment of the load-displacement curve [14,16]. The fracture toughness (*K_C_*) can be evaluated by using the following simple equation [14]

(2) $$K_{c}=\gamma \;\frac{P_{c}}{R^{3/2}}$$

where *K_c_* is the fracture toughness (MPa.m^1/2^), *P_c_* is the critical load at failure (mN) and *R* the pillar radius (µm). *γ* is a dimensionless coefficient and has been calculated for a wide range of materials properties in a recent paper [16]. It is worth noting that the *γ* coefficient contains the influences of elastic and plastic properties and is consequently material specific. The usefulness of Equation (2) lies in its simplicity, as both the critical load and pillar radius are easily measured quantities. Recent papers have demonstrated the applicability of the pillar indentation splitting method for a wide range of material properties, which includes most ceramic materials [17,18,19].

The second method uses atomic force microscopy (AFM) to measure the crack opening displacements (COD) after nanoindentation. Irwin’s near field solution was then applied to evaluate the mode I crack tip toughness as first introduced by Rödel et al. [15] by means of scanning electron microscopy. Additionally, indentations are performed on a wafer of spinel LiMn_2_O_4_ with its top surface parallel to the {111} plane. This highly-oriented crystal enabled reproducible crack patterns around the indents and Anstis solution for half-penny cracks [9] was used to evaluate the fracture toughness and is also used as a reference in this work. Crack evolution below the surface of the indent was also observed for the wafer using the FIB cross sections. 

Lastly, both microscale techniques are compared based on their merits and demerits and recommendations are provided on the use of suitable technique for the fracture toughness assessment of this challenging material. 

## 2. Materials and Methods 

### 2.1. Sample Preparation 

Active particles of LiMn_2_O_4_ based cathode material extracted from commercial cells and cycled three times at one C-rate from 2.5 to 4.2 V. These cathode materials were prepared for nanoindentation as described in the previous work [20]. A wafer of {111}-oriented lithium manganese (III, IV) oxide was prepared using a wafer of {111}-oriented manganese (II) monoxide as precursor (SurfaceNet GmbH, Rheine, Germany). The preparation method developed by Kitta et al. [21] was used.

### 2.2. Pillar Splitting Experiments

Fabrication of the micro-pillars were performed using the focused ion beam (FIB) procedure based on the ring-core milling approach developed by some of the authors [22,23]. The milling was performed in a single outer to inner pass using the FEI Helios NanoLab 600 at a current of 0.92 nA. At least five pillars were milled to an aspect ratio (h/d) of >1.2, where h is the pillar height and d is the top diameter. It has been shown previously [22] that this geometrical design provides complete residual stress relaxation in the upper part of the pillar. It is worth highlighting that using the correct combination of current and dwell time; a single pillar can be milled within 10 minutes. Pillars were only milled on the particles that are wide and deep enough to accommodate a 5 µm pillar. Special care was taken to avoid the porous particles. All pillars were tested using a Berkovich indenter on a Keysight G200 nanoindenter at a constant strain rate of 0.05 s^−1^ and an indentation depth set to 400 nm into the top surface. The instrument frame stiffness and indenter area function were calibrated before and after testing on a certified fused silica reference sample. The continuous stiffness measurement (CSM) mode was turned off during the tests.

### 2.3. Crack Opening Displacement Measurement 

**Figure 1:**
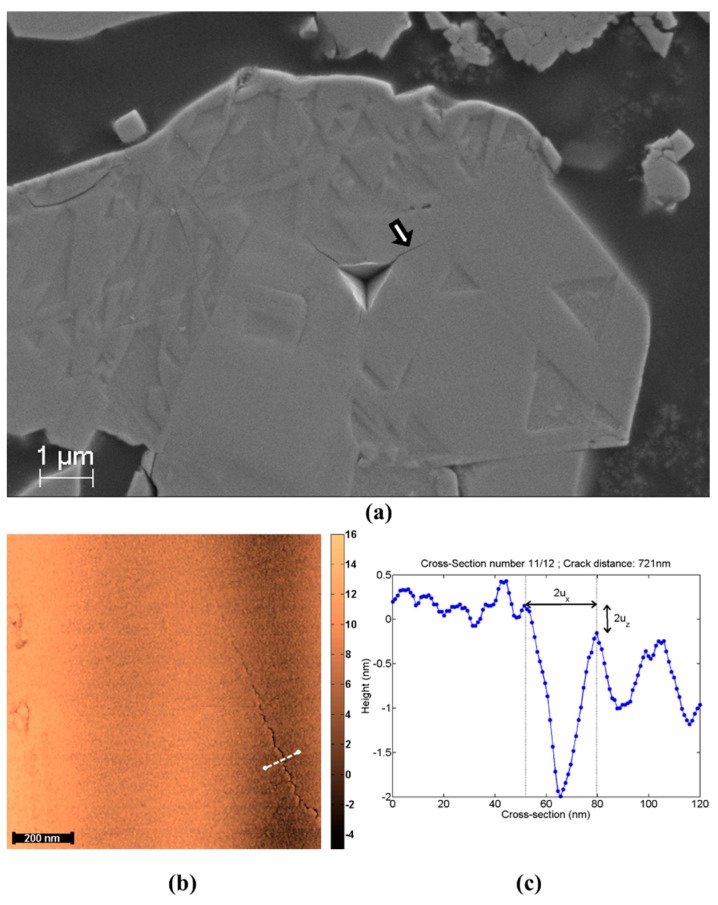
(**a**) SEM micrograph of a cube-corner indent in a LiMn_2_O_4_ particle. The cracks do not propagate straight from the corners of the indent. They are also not longer than the indent; the arrow indicates the crack mapped by means of AFM and visible in (**b**,**c**) is the cross-section (indicated by a white line in (**b**)) used to estimate *u_x_* for *x* = 721 nm.

A thin layer (~1 nm) of Pt/Pd alloy was sputter coated on each sample for easy imaging by scanning electron microscope (SEM). The samples were indented with cube corner tips down to 400 nm without continuous stiffness measurement and with a strain rate target of 0.05 s^−1^, leaving an indentation print about 600 nm wide. The SEM was used to determine the suitability of the cracks for COD measurements (see Figure 1a). The cracks were selected if they were long enough to be mapped and did not grow too close to a particle edge or a defect. Tapping mode atomic force microscope (Dimension 3100) using very sharp tips imaged each crack (TESP-SS, Bruker, 42 N/m, 320 kHz, 5 nm max radius) (see Figure 1b). The challenge was not to smooth the tips while scanning as the stiff particle edges can easily damage them. Approach and scan were done using a very small initial force set point (2%) in order to find the cracks. Once a crack was found, the force was increased until trace and retrace lines were similar. TGX1 test grating samples (NT-MDT) were used before and after measurements to characterize the AFM tip sharpness using the same procedure. In order to minimize sub-critical crack growth between crack formation and COD measurement, indentations were carried out in the early morning and the rest of the procedure was carried out within one day, sometimes extending to the next day. For a crack along the x-axis and its tip located at (*x* = 0, *y* = 0), Irwin describes the crack displacement as follows [24]:

(3) $$u_{x}\left(X\right)=\{\frac{\left(1-v^{2}\right)K_{IC}}{\begin{array}{c} E\\ 0,\;X<0 \end{array}}\sqrt{\frac{8X}{\pi }}\;,\;X\;\geq 0$$

where *u_x_*(*X*) represents the near-tip crack opening displacement at position (*x* = *X*, *y* = 0), *E* is the elastic modulus, *ν* represents the Poisson’s ratio, and *K_IC_* is the mode I fracture toughness. For each image, *u_x_* was measured on 12 cross-sections perpendicular to the crack for different distances *X* to the crack tip. Special care was taken to avoid reverse tip imaging at the crack walls. *K_IC_* was calculated by measuring the slopes of *X* vs. *u_x_* and inputting the value into Equation (3). Measurement reliability is assessed by the correlation coefficient R^2^ of the linear regression. 

## 3. Results

### 3.1. Crack Length Measurement

The fracture toughness measurement using the classical Anstis solution for half-penny cracks is highlighted in Equation (1) and largely relies on the configuration of crack pattern. This is the most widely used method for fracture toughness calculations. For the case of LiMn_2_O_4_ particles (Figure 1a), the configuration of the crack pattern does not allow the application of the crack-length measurement method traditionally used after indentation [25]. However, the cracking pattern of the reference wafer sample (pictured in Figure 4a) seems to allow such measurements. Using Anstis solution for half-penny crack [9] and E and H values of 95.70 GPa and 6.70 GPa respectively, a fracture toughness of 0.33 ± 0.07 MPa.m^1/2^ was calculated for the wafer sample. It is worth mentioning that a α value of 0.0569 was used for the calculation of fracture toughness as proposed by Jang and Pharr [13] for the cube corner indenters. One might wonder at this point that the coefficient value of 0.0569 was originally proposed for Si and Ge material. The usability of this coefficient value is justified by comparing the E/H ratio of Si and the wafer material used in this study. Both Si and wafer material have an E/H ratio of 14.60 and 14.30, respectively. This value of fracture toughness (0.33 MPa.m^1/2^) will be used as a reference and is not further detailed in this work.

### 3.2. Pillar Compression

**Figure 2:**
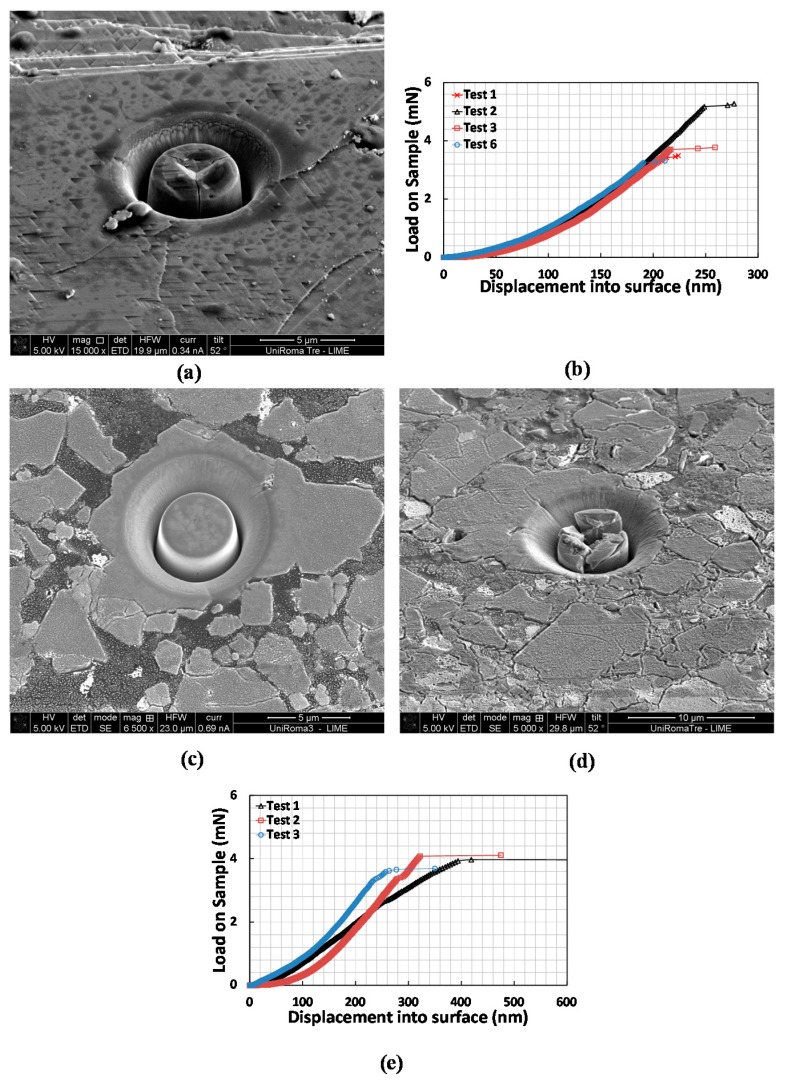
(**a**) SEM micrograph of a split pillars on the wafer and (**b**) a representative load-displacement curve highlighting the critical splitting load by a pop-in event; (**c**) SEM micrograph of a pillar on commercial LiMn_2_O_4_ particle before and (**d**) after splitting; (**e**) Representative pillar splitting data obtained on the commercial LiMn_2_O_4_ particles.

Figure 2a shows a SEM micrograph of a pillar after splitting along with the load displacement curve on the reference wafer sample (Figure 2b). The fracture toughness was calculated using Equation (2) and the *γ* value used for this LiMn_2_O_4_ sample is 0.25, obtained by finite element modeling (FEM) as described in a previous study [14], assuming a value of 95.73 GPa for the elastic modulus and 6.71 GPa for the hardness [26]. Regarding the tests on the real commercial cathode samples, an example of a pillar before and after splitting (discharged fresh cell) is reported in Figure 2c,d. Results from splitting experiments on a series of FIB-milled pillars are shown on Figure 2e. Using the elastic modulus of 86.67 GPa and a hardness of 6.95 as reported in a previous paper [26], a *γ* coefficient of 0.22 was calculated. Note that this value also includes the correction, obtained through CZ-FEM, for the effects coming from the compliant polymer substrate. A critical failure load of 3.90 ± 0.22 mN gives a toughness value of 0.24 ± 0.01 MPa.m^1/2^, which is in very good agreement with the estimations obtained on the LiMn_2_O_4_ reference wafer. Table 1 summarizes the results for both samples.

### 3.3. Crack Opening Displacement 

**Figure 3:**
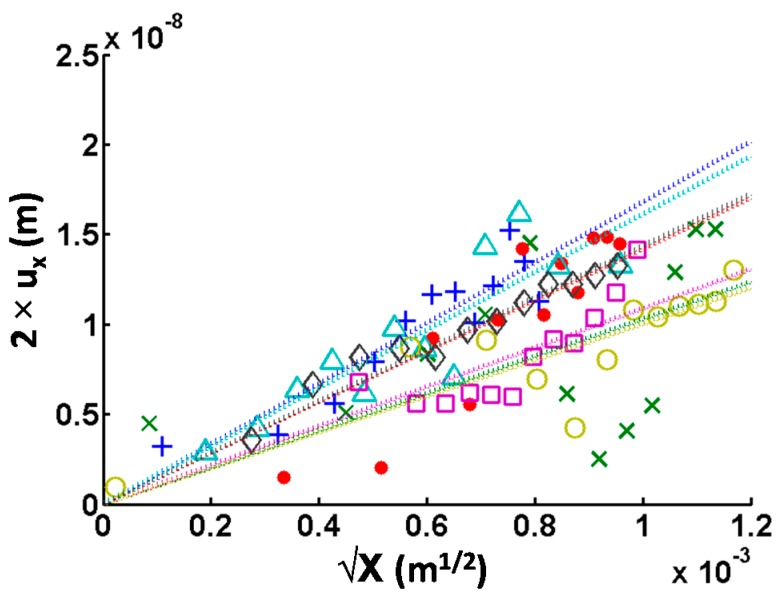
Crack Opening Displacement (2 × *u_x_*) versus square root of distance to crack tip (√X) for seven different cracks. Each plot is fitted with a linear function forced to zero (dotted lines). Colors of markers from the experimental measurements correspond to colors of fitting lines.

**Figure 4:**
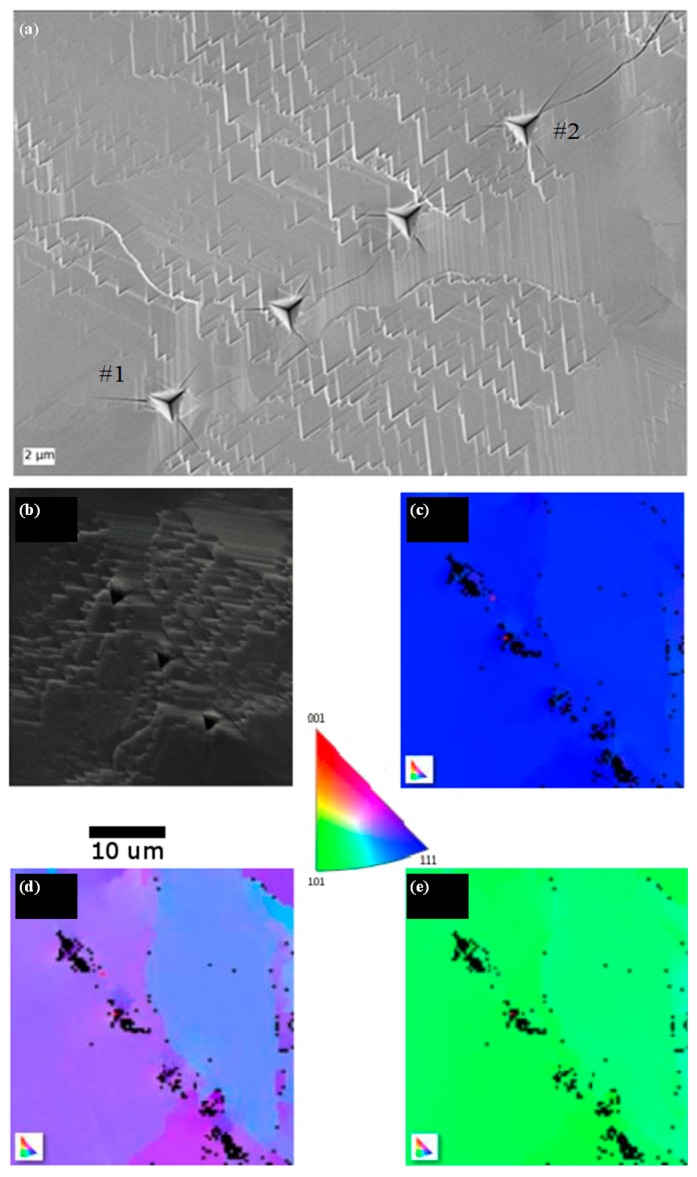
(**a**) SEM micrograph of four 400-nm deep indents performed by depth control on the wafer sample. Cracks of particle labeled #1 and #2 were observed by FIB cross-sections; (**b**) Secondary electron image of the mapped area; (**c**) Z direction; (**d**) Y direction; and (**e**) X direction. Color-coding indicated in the center.

The COD and the height difference versus the distance to the crack tips of seven cracks are plotted in Figure 3. It is evident that not all measurements present the same quality; some are relatively linear while others are very irregular. This is due to the roughness of the particles, such as scratches from polishing, leading to imprecisions. Experimental measurements were fitted with linear functions forced to zero. The crack tips were previously positioned from the AFM error images. The slope is inserted in Equation (3) to find *K_IC_* using an elastic modulus of 90 GPa [20] and a Poisson’s ratio of 0.3. The quality of the measurements was determined from the coefficient of determination R^2^. Weighted means were applied to obtain a quantitative value, the weights being the R^2^ of each fit. Table 2 summarizes all the results. For reference, the same method was used for the cracks on wafer (Figure 4a) and a crack-tip toughness of about 0.7 MPa.m^1/2^ was found.

### 3.4. Crack Orientation 

**Figure 5:**
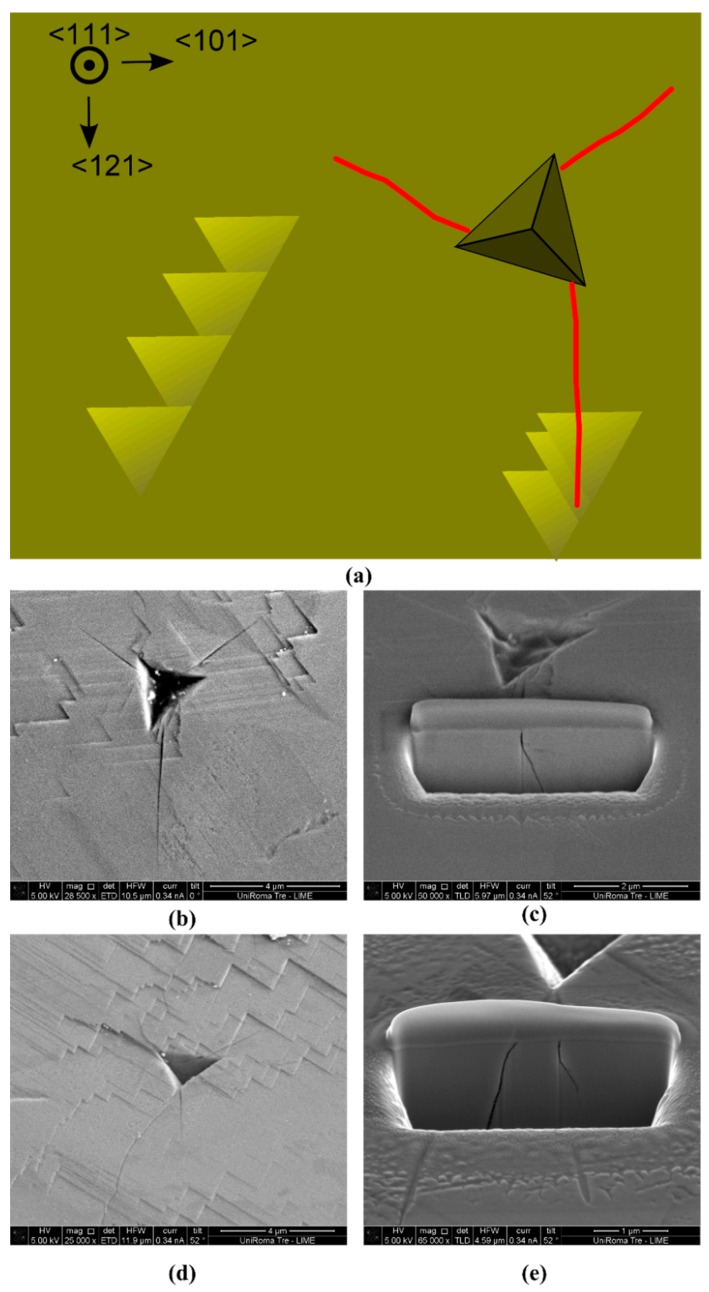
(**a**) Schematics of the wafer. Yellow areas correspond to the wafer materials where the triangles represent the patterns observed on Figure 4a, the darker triangle represents an indent print with a ‘random’ rotation and the red lines represent typical cracks, growing perpendicularly to the edge of the pattern triangles. (**b**,**d**) SEM images of two 400 nm deep indents before and (**c**,**e**) after FIB milling. The first indent (**b**,**c**), labeled #1 in Figure 4a, produced a reproducible crack pattern where three cracks formed perpendicularly (or 30°) to the edges of the triangular pattern during crystal growth, regardless of the indenting tip rotation. The second indent (**d**,**e**), labeled #2 in Figure 4a, produced two cracks at its corner, one similar to the previous one and another one which is much longer and certainly due to defects. Under the surface (**c**–**e**), the cracks grew perpendicularly to the top surface for about 100 nm before deviating of an angle of 30–40°.

Figure 4a is an SEM image of the wafer after indentation. The 60° facet edges indicate the highly-oriented crystal as previously obtained (see Reference [21]). It is not a single crystal as can be seen from EBSD measurements (Figure 4b–e) but all the grains have their top surface parallel to the {111} plane and have only a little misorientation. The edges of the triangular pattern are perpendicular to the <121> direction. All the indents formed cracks growing along the <121> direction (Figure 4a), hence forming {101} planes if perpendicular to the top surface. The cracks always grow perpendicularly to the edges of the triangular pattern regardless of the orientation of the indenting diamond tip. Figure 5 schematically depicts this phenomenon along with FIB cross-sections to see the cracks development below the surface. They indicate that the cracks grew first perpendicularly to the top surface, opening {101} plane within a depth of 100 nm. Then they deviate at an angle of 30° to 40°. There are two possible explanations for this deviation. Another material could be present below the spinel material. In fact, X-ray diffraction measurements published in a previous work [26] showed that Bixbyite Mn_2_O_3_ could be present and it is possible that there is a deficit of oxygen ions between the cubic MnO substrate and the spinel LiMn_2_O_4_ top surface. Otherwise, only the spinel material could be present and the propagation deviates because surface formation is easier in this new plane. 

## 4. Discussion

### 4.1. Reliability of the Methods

#### 4.1.1. Pillar Splitting Method

A critical analysis of reliability of the pillar splitting method along with its applicability for a series of reference coating and bulk materials is presented in previous papers [14,16]. The main advantage of this technique is that the effect of FIB damage on the measurements is almost negligible. This is because the crack nucleation and growth usually happens within the core volume of the pillars, whilst the FIB damage is only present in the very 10–100 nm at the pillars edges. In comparison with the COD method, the pillar splitting method seems more reliable as it is easier to set-up experimentally and provides highly reproducible pillar splitting loads. Another advantage of this technique over the conventional methods which used the nanoindentation technique for fracture toughness assessment is that there is no need to measure the crack length, hence not only significantly enhancing the test time but also favoring less experimental hazard. One particular challenge regarding the lithium-based composite electrodes is the influence of the surrounding compliant substrate. This issue is addressed in a recent paper [16], which shows that the effect of complaint matrix on the critical splitting load is ~11% in the worst case scenario and can be corrected by evaluating specific values of γ coefficient. The calculated value of fracture toughness (0.27 ± 0.06 MPa.m^1/2^ for wafer and 0.24 ± 0.01 MPa.m^1/2^ for particles) using pillar splitting method already include the substrate corrected coefficient values. These values of fracture toughness are in very good agreement with the Anstis half-penny crack method, which gives a value of 0.33 ± 0.08 MPa.m^1/2^. There is no quantitative report of fracture toughness on these challenging materials in literature with the exception of one paper recently published by the authors [17]. In that article, authors studied the change in fracture toughness vs. the state of charge and observed a decrease in fracture toughness (0.49 to 0.26 MPa.m^1/2^) as the state of charge increases. Finally, these values of fracture toughness are in very good agreement with the reported values of similar cathode materials, e.g., Wolfenstein et al. reported the fracture toughness of Li-olivine cathodes (LiCoPO_4_) between 0.4–0.5 MPa.m^1/2^ [27]. This further confirms that the pillar splitting technique is a more reliable method for measuring the fracture toughness of LiMn_2_O_4_ particles. 

#### 4.1.2. Crack Opening Displacement

**Figure 6:**
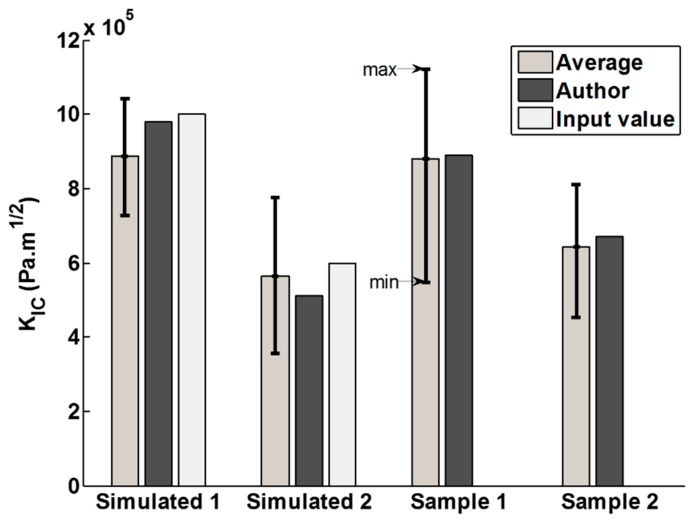
Mode I fracture toughness values obtained from the group experiment. The first set of bars show the average toughness measured by the group. The error bars associated with it indicate the highest and lowest values obtained from the group. The darker set of bars show the values obtained by the first author. The lighter set of bars show the values inputted to simulate the surfaces. Sample **1** and sample **2** correspond to ⎝ and © in Table 2 and Figure 3.

For COD measurements, it is difficult to apply higher loads without destroying the particles, rendering measurements impossible. Inevitably, the crack lengths come close to the typical size of an indent as shown in Figure 1a. It is important to highlight that the strong hypothesis of Irwin’s near-field solution is that the crack walls are traction free [24]. This could be the main reason for the systematic error in COD measurements, because some residual stress may be present in the particles due to their processing. On the contrary, residual stresses are fully relieved in the case of pillar geometry as demonstrated in previous papers [22,23]. A second issue is the measurement uncertainty coupled with user’s interpretation of AFM measurements. It is difficult to decipher inverse AFM tip imaging from crack wall imaging as the measured vertical displacements *u_z_* have the same order of magnitude as the surface roughness. This can be observed from real measurements in Figure 1c. This behavior was simulated using Equation (3), tip radius effects and artificial images were generated which are then interpreted by the group of scientists. Figure 6a highlights the mode I fracture toughness values obtained from the group experiment. It can be noticed how close the computed values are to the input values in the case of the simulated cracks. Yet a user’s interpretation can drastically change the measurement from simple to double, as is the case for sample 1. Overall, the averaged values from the group approach the author’s measurements, which shows that most users interpreted the COD the same way. These results show that the COD measurements using Irwin’s near field theory are not the reliable method to quantify fracture properties of micrometric particles particularly because of the systematic error associated with the plastic zone.

## 5. Conclusions

Fracture toughness of commercial LiMn_2_O_4_ particles embedded in a polymer matrix are evaluated using two micro-scale techniques. Pillar splitting method gives a fracture toughness value of about 0.24 MPa.m^1/2^, while crack-opening displacement gives a value of ~0.85 MPa.m^1/2^. The first method appeared to be more reliable for determining the fracture toughness properties of these materials because of the ease of experimental setup, well-defined pillar geometry, simplicity, and reproducibility of results. In the case of the second method, the size of the particles does not allow crack growth to a size where stress-free crack walls and elastic–brittle theory can be considered for COD measurement. Concerning materials properties, this ceramic seems very brittle as its toughness lies in the lower range of ceramics toughness (below 1 MPa.m^1/2^). Additional tests on an oriented LiMn_2_O_4_ wafer showed that fracturing is also very dependent on the crystal orientation of the indented grains as the cracks always grow in the <121> direction on {111}-oriented surfaces, with a possible favorite cleavage plane being {101}. This further point to the possibility of producing engineered structure and/or the particle shapes to reduce brittleness.

## Figures and Tables

**Table 1:** Results summary for pillar compression tests on LiMn_2_O_4_ wafer and particles.

Parameter	Wafer	Particles
E-modulus, E (GPa), [26]	95.73 ± 3.93	86.67 ± 11.29
FE Poisson’s ratio, *ν*	0.25	0.25
Hardness, H (GPa), [26]	6.71 ± 0.44	6.95 ± 0.76
Substrate corrected finite element *γ* (Equation (1))	0.25	0.22
Experimental pillar radius, R (µm)	2.36 ± 0.10	2.36 ± 0.10
Experimental instability load, *P_c_* (mN)	3.88 ± 0.85	3.90 ± 0.22
Fracture toughness, *K_c_* (MPa.m^1/2^)	0.27 ± 0.06	0.24 ± 0.01

**Table 2:** Mode I fracture toughness values of seven different cracks on LiMn_2_O_4_ particles using crack opening displacement technique.

Sample	*K_IC_* (MPa.m^1/2^)	R^2^
+	1.00	0.84
⎝	0.89	0.95
⌈	0.62	0.69
©	0.67	0.66
⌠	0.99	0.77
│	0.88	0.70
×	0.64	0.13
Weighted means	0.81% ± 18%	N/A
